# The dual functions of α-tubulin acetylation in cellular apoptosis and autophage induced by tanespimycin in lung cancer cells

**DOI:** 10.1186/s12935-020-01453-y

**Published:** 2020-08-05

**Authors:** Qilin Wang, Xiangguo Liu

**Affiliations:** 1 Liaocheng University School of Life Sciences, No. 1, Hunan Road, Dongchangfu District, Liaocheng, 252059 People’s Republic of China; 2 Shandong University School of Life Sciences, 72 Binhai RD, Qingdao, 266237 People’s Republic of China

**Keywords:** α-tubulin acetylation, Tanespimycin, Cellular apoptosis, Autophage, Hsp90

## Abstract

**Background:**

Reversible acetylation of α-tubulin has been implicated in modulating microtuble structures and functions, which may subsequently involve in cellular apoptosis and autophage. But how to trigger apoptosis or autophage at what level of acetylated α-tubulin (Ac-α-tubulin) are not known. This study aims to demonstrate the dual functions and molecular mechanisms of α-tubulin acetylation in cellular apoptosis and autophage induced by tanespimycin in Calu-1 cells simultaneously.

**Methods:**

Calu-1 cells were treated with tanespimycin alone or combined administrations of different agents (including TSA, Docetaxel, Rapamycin, 3-MA and Z-vad) respectively and cell lysates were prepared to detect the given proteins by Western Blot. The cell survival was observed by inverted phase contrast microscope and estimated by SRB assay. HDAC6, TAT1 and Hsp90α/β proteins were knocked down by siRNA technique.

**Results:**

By combination administration of tanespimycin with TSA or Docetaxel, the expression of Ac-α-tubulin and cellular apoptosis were enhanced markedly. While combination of tanespimycin and Rapamycin, α-tubulin acetylation and apoptosis were inhibited, but LC3B-II expression was facilitated substantially. When tanespimycin was combined with autophage inhibitor 3-MA, α-tubulin acetylation elevation was apparently, but LC3B-II was attenuated. Apoptosis inhibitor Z-vad blocked partially Caspases activation induced by tanespimycin, but failed to hinder α-tubulin acetylation elevation. According to results of RNA interference, acetyltransferase TAT1, deacetylase HDAC6 and Hsp90 modulated the expression level of α-tubulin acetylation.

**Conclusion:**

We have elucidated that acetylation of α-tubulin induced by tanespimycin has dual functions in cellular apoptosis and autophage and the level of α-tubulin acetylation reaches a degree Calu-1 cells undergo cell apoptosis rather than autophage, implying that the level of acetylated α-tubulin may determine cell fate for survival or apoptosis.

## Background

With the entry of tanespimycin into clinical phage II and III, more and more studies have sought to investigate the effect of combined administration of tanespimycin and other anticancer drugs in different cancer cells [1–3]. Tanespimycin is a specific inhibitor of Hsp90 and disrupts Hsp90 molecular chaperone activity and consequently promotes variety of Hsp90 client protein degradation. It has been investigated that tanespimycin promoted removal of mutant androgen receptor by autophagic degradation pathway in spinal and bulbar muscular atrophy [4]. In another study, pharmacological inhibition of Hsp90 by tanespimycin potentiated cellular apoptosis [5]. Recently tanespimycin has been reported in literatures to induce not only cell autophage but apoptosis in different cell lines [6, 7]. Therefore, studies on tanespimycin in cancer cell apoptosis, autophage, clinical therapy and so on have increased [1–9]. For example, combination of tanespimycin and PI3K/mTOR inhibitor NVP-BEZ235 had synergistic anti-tumor effect on human melanoma [2]. In another reported research, tanespimycin induced apoptosis of myogenic cells through activation of the intrinsic pathway [8]. Furthermore, tanespimycin has been testified as a promising agent for multiple myeloma therapy [9]. These results show that cellular apoptosis or autophage induced by tanespimycin may be some correlation.

Now we all know that the main pathway for protein degradation in apoptosis is the ubiquitin–proteasome system (UPS) [10]. UPS includes multi-protein proteolytic complex that degrades short-lived proteins, such as denatured proteins, misfolded proteins and some signal modulating proteins, all which are marked by the ubiquitin/ubiquitins. Deacetylase HDAC6 is reportedly involved in transportation and clearance of misfolded proteins [11, 12]. Alternatively, HDAC6 mediates and coordinates the major pathways for degradation of misfolded and aggregated proteins dependent on molecular chaperone [13].

α-Tubulin and Hsp90 are two substrates of deacetylase HDAC6, and they will be acetylated when HDAC6 is inhibited [14, 15]. In addition, HDAC6 is also the substrate of Hsp90 reported in other study [16], which means that Hsp90 inhibition will influence the expression level of HDAC6 and consequently the level of acetylated α-tubulin. α-Tubulin is an important component of microtubules and so acetylation of α-tubulin can modulate the stability and dynamic activity of microtubules, which subsequently regulate microtubule properties, such as cell shape maintenance, cell mitosis, cell meiosis, intracellular trafficking, and so much the cell fate for survival or apoptosis [17]. Therefore, the acetylation extent of α-tubulin in cell apoptosis exerts important roles [18]. It is well known that α-tubulin is acetylated or deacetylated on the ε-amino of lysine residue at position 40 by α-tubulin acetyltransferase MEC-17/TAT1 or deacetylase HDAC6 [14, 19]. HDAC6 has been investigated as the target of anticancer drugs for the treatment of high metastasis and advanced malignancies [20], which may correlate with the regulatory role of α-tubulin acetylation. That is to say, deacetylase HDAC6 plays an important effect on the microtubule network functions and properties by affecting the acetylation of α-tubulin [13, 21–23].

In addition, acetylation of α-tubulin and HDAC6 inhibition enhance autophagy in non-small cell lung cancer [18]. HDAC6 may influence the autophagosome-lysosome fusion and furthermore affect autophage [24, 25]. During autophagesome formation endogenous LC3 (microtubule-associated protein 1 light chain 3) is processed into a cytosolic isoform LC3B-I, which is converted into membrane-bound LC3B-II. The amounts of LC3B-II are correlated with the number of autophagesomes and LC3B-II is also identified as a specific marker for autophagesome [26, 27]. In our study, we discovered that Hsp90 inhibition by tanespimycin induced cellular apoptosis and autophage simultaneously accompanying the different expression levels of acetylated α-tubulin and LC3B-II. So we want to know whether the cellular apoptosis and autophage induced by tanespimycin determines the cell fate in consistent or not, and whether there is the convergence point of acetylated α-tubulin level, from which leads to cell death and the other to cell survival. At present the correlation of α-tubulin acetylation between apoptosis and autophage is not well known and even controversal. So our study tends to explore the functions of α-tubulin acetylation between autophage and cell apoptosis induced by tanespimycin in Calu-1 cells of lung cancer.

Lung cancer is one of the malignant tumors with the fastest increasing morbidity and mortality and has a great threat to people’s life. Our study team have been investigating the mechanism of apoptosis and autophage in lung cancer cells and screening anti-tumor drugs. In the lung cancer cell lines, Calu-1 is the epithelial transformed cells derived from metastatic pleura site and contains the ras (K-ras) oncogene. In nude mice Calu-1 can form epidermoid carcinomas. This cell line also contains human papillomavirus type 18 (HPV-18) DNA sequence and expresses HPV18 RNA. Calu-1 cell line is easy to be cultured and the morphological changes of the cell are easy to be observed, and we choose this cell line in this study.

## Materials and methods

### Reagents and antibodies

Tanespimycin was provided by LC Laboratories (Woburn, MA). Docetaxel was purchased from American Radiolabeled Chemicals, Inc. (St. Louis) and Rapamycin, TSA, 3-MA and Z-vad reagents were from Cell Signaling Technology (Danvers, MA). Each of these compounds was dissolved in dimethyl sulfoxide (DMSO) at a given concentration and aliquots were stored at − 20 °C. Stock solutions were diluted to the final appropriate concentrations just before use.

Mouse anti-caspase-3 monoclonal antibody was purchased from Imegenex (San Diego, CA). Mouse anti-caspase-8, rabbit anti-caspase-9, rabbit anti-LC3B-II and rabbit anti-poly (ADP-ribose) polymerase (PARP) monoclonal antibodies were purchased from Cell Signaling (Cell Signaling Technology, Danvers, MA). Mouse anti-Hsp90α and Rabbit anti-Hsp90β monoclonal antibodies were from Abcam (Cambridge, UK). Mouse anti-HDAC6, goat anti-TAT1 and mouse anti-β-actin polyclonal antibodies were from Sigma (Santa Cruz Biotechnology, Santa Cruz, CA). Mouse anti-Ac-α-tubulin and α-tubulin polyclonal antibodies were from Cell Signaling (Cell Signaling Technology, Danvers, MA).

### Cell lines, cell culture and transfection

Calu-1 cells used in this study were provided by the American Type Culture Collection (ATCC, Manassas, VA) and were cultured in RPMI 1640 (Gibco, Grand Island, NY) with 5% fetal bovine serum in a 95% air and humidified atmosphere of 5% CO_2_ at 37 °C. Calu-1 cells were transfected with given plasmids by using the FuGene HD reagent (Roche Molecular Biochemicals).

### Cell survival assay

First, Calu-1 cells in good conditions were seeded in 96-well plate with a moderate amount in 100 μL culture medium per well. On the second day, the tested compounds or drugs were mixed in and the total volume was 200 μL/per well. Cells were treated for definite time and then cell survival was estimated by sulforhodamine B (SRB) assay.

### Western blot analysis

The preparation of whole-cells protein lysates and the related analysis of western blot results were described in our previous published paper in reference [28].

### Knockdown of Hsp90, TAT1 and HDAC6 expression with siRNAs

siRNAs were synthesized by GenePharma (Shanghai, China) and the interference experiments of siRNAs were conducted as previously described in our published paper [28]. Hsp90α/β siRNA target sequences were described before [29], and other siRNAs target sequences were described as follows.

Control siRNA oligos was: 5′-UUCUCCGAACGUGUCACGUTT-3′;

TAT1 siRNA oligos was: 5′-GGGAAACUCACCAGAACGA-3′.

HDAC6 siRNA oligos was: 5′-CCAAUCUAGCGGAGGUAAA -3′.

## Results

### Tanespimycin induces upregulation of Ac-α-tubulin and LC3B-II

**Fig. 1:**
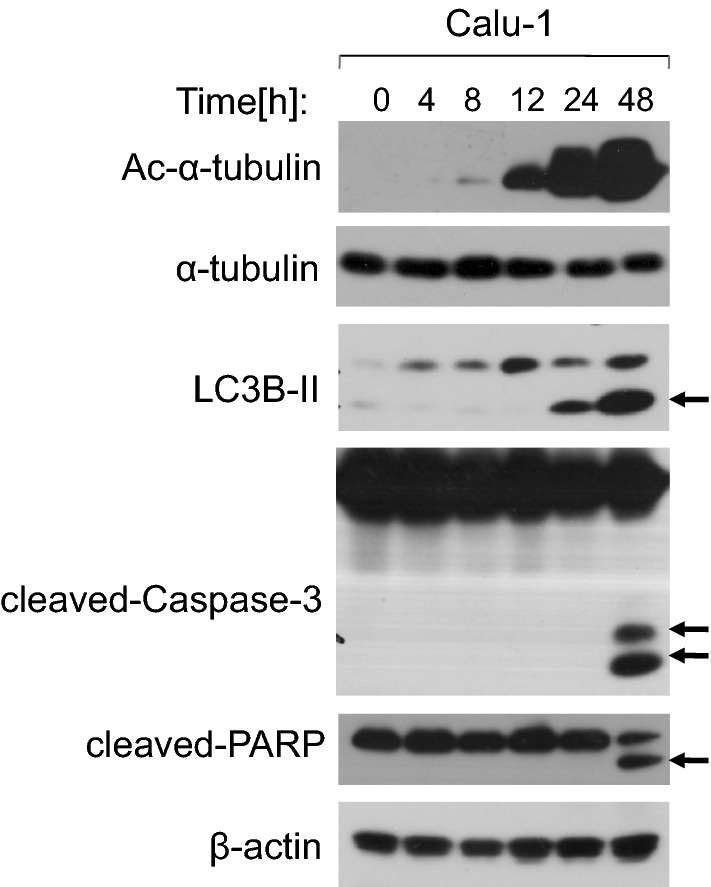
Tanespimycin induces upregulation of Ac-α-tubulin and LC3B-II. Calu-1 cells were treated with 1.0 μM tanespimycin for the given time (0, 4, 8, 12, 24, 48 h), and then the cells were subjected to preparation of whole-cell protein lysates and the given proteins (Ac-α-tubulin, α-tubulin, LC3B-II, cleaved-Caspase-3, cleaved-PARP and β-actin) were detected by Western blot analysis

In order to study the function of α-tubulin acetylation in cell apoptosis and autophage, the time-course experiments in Calu-1 cancer cells were performed firstly. Our study results showed that when Calu-1 cells were exposed to tanespimycin (1.0 μM for 0, 4, 8, 12, 24, 48 h), α-tubulin acetylation (Ac-α-tubulin) and cell apoptosis (evidenced by Caspase-3 and PARP activation) were induced apparently at 48 h, and meanwhile LC3B-II expression was increased obviously (Fig. 1). LC3B-II is the marker of autophage and its amounts correlates with the quantity of autophagesomes [26, 27]. Therefore, according to above results it is concluded that tanespimycin induces autophage and apoptosis in Calu-1 cells, and α-tubulin acetylation may be correlated with not only apoptosis but autophage.

**Fig. 2:**
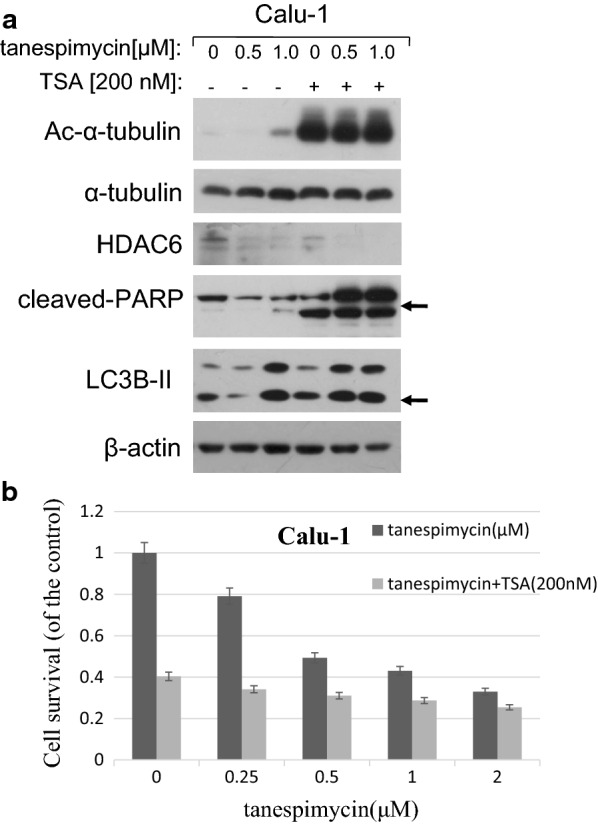
TSA enhances Ac-α-tubulin upregulation and cell apoptosis induced by tanespimycin. **a** Calu-1 cells were treated with the given concentrations of tanespimycin (0, 0.5, 1.0 μM) or combination with TSA (200 nM) for 48 h, and then the cells were subjected to preparation of whole-cell protein lysates and the given proteins (Ac-α-tubulin, α-tubulin, HDAC6, PARP, LC3B-II and β-actin) were detected by Western blot analysis. **b** Calu-1 cells were seeded in 96-well plate and on the 2nd day treated with the given concentrations of tanespimycin (0, 0.25, 0.5, 1.0, 2.0 μM) or combination with TSA (200 nM) for 48 h. Cell number was estimated by SRB assay for calculation of cell survival. Points, mean of four replicate determinations; bars, SD

α-Tubulin is a well known substrate of deacetylase HDAC6. If HDAC6 is inhibited, the expression of α-tubulin acetylation is increased. Hence, in order to further testify the function of α-tubulin acetylation induced by tanespimycin in autophage and apoptosis, tanespimycin was combined with TSA, an inhibitor of HDAC6. Our experiments demonstrated that tanespimycin alone in Calu-1 cells induced HDAC6 decrease, but apparently promoted the expressions of α-tubulin acetylation and LC3B-II (Fig. 2a), implying that α-tubulin acetylation had correlation with autophage. While tanespimycin was combined with TSA, the expression levels of α-tubulin acetylation and PARP activation were increased substantially, and HDAC6 was reduced markedly, which means that cell fate for death is predominant (Fig. 2a). From another aspect, these results showed that HDAC6 decrease or α-tubulin acetylation was also involved in apoptosis (evidenced by PARP activation). Though the expression level of LC3B-II is not changed apparently (compared to tanespimycin treatment at 1.0 μM) (Fig. 2a), the ratio of cell survival decreased more in combination of tanespimycin and TSA by SRB detection (Fig. 2b). Taken together, acetylation of α-tubulin induced by tanespimycin is involved in apoptosis and autophage in lung cancer cells.

### Docetaxel promotes Ac-α-tubulin upregulation and inhibits LC3B-II augment induced by tanespimycin

**Fig. 3:**
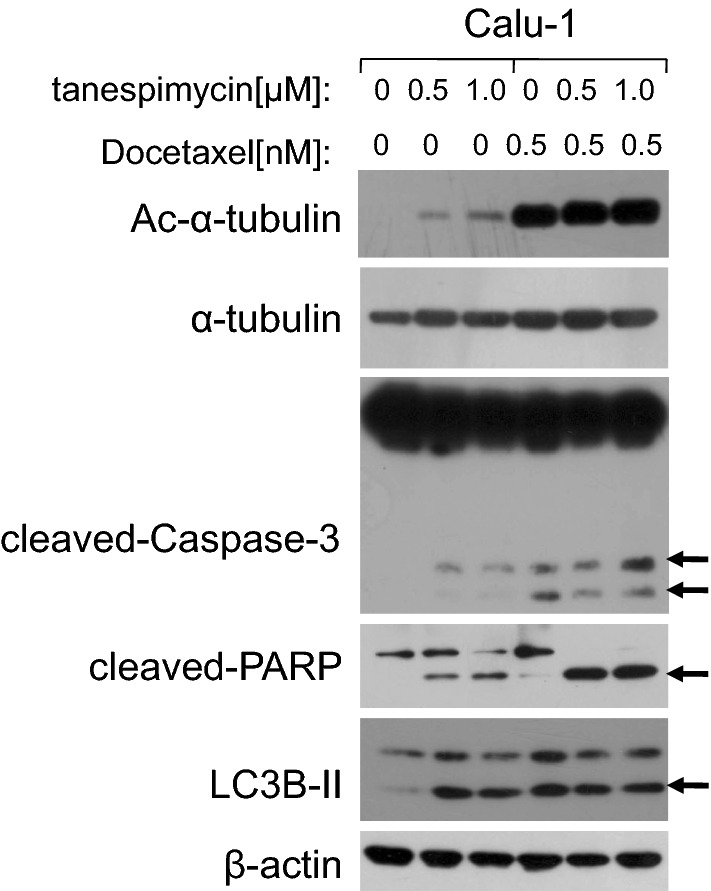
Docetaxel promotes Ac-α-tubulin upregulation and inhibits LC3B-II augment induced by tanespimycin. Calu-1 cells were treated with the given concentrations of tanespimycin (0, 0.5, 1.0 μM) or combination with Docetaxel (0.5 nM) for 48 h, and then the cells were subjected to preparation of whole-cell protein lysates and the given proteins (Ac-α-tubulin, α-tubulin, cleaved-Caspase-3, cleaved-PARP, LC3B-II and β-actin) were detected by Western blot analysis

Docetaxel is a microtubule stabilizer and an effective anti-tumor drug in treatment of lung cancer and breast cancer. When cells are treated with Docetaxel, the expression of Ac-α-tubulin will increase and result in cell division blockage and apoptosis induction. In our study it was discovered that tanespimycin alone induced activiation of Caspase-3 and PARP, and also LC3B-II expression, suggesting that cells experienced apoptosis or tanespimycin induced autophage in Caspase-dependent way. Docetaxel alone also induced autophage at the concentration of 0.5 nM compared to the control evidenced by activation of LC3B-II (Fig. 3). When combination of tanespimycin and Docetaxel, the expression of Ac-α-tubulin was increased further in Calu-1 cells, but LC3B-II was attenuated slightly which implied that cell apoptosis was enhanced while autophage was inhibited. In a word, there would be existence of competition of cell apoptosis and autophage in Calu-1 cells treated with tanespimycin, and acetylation of α-tubulin was both involved in cell apoptosis and autophage, which was determined by the acetylation level of α-tubulin.

### Rapamycin inhibits cell apoptosis and α-tubulin acetylation induced by tanespimycin

**Fig. 4:**
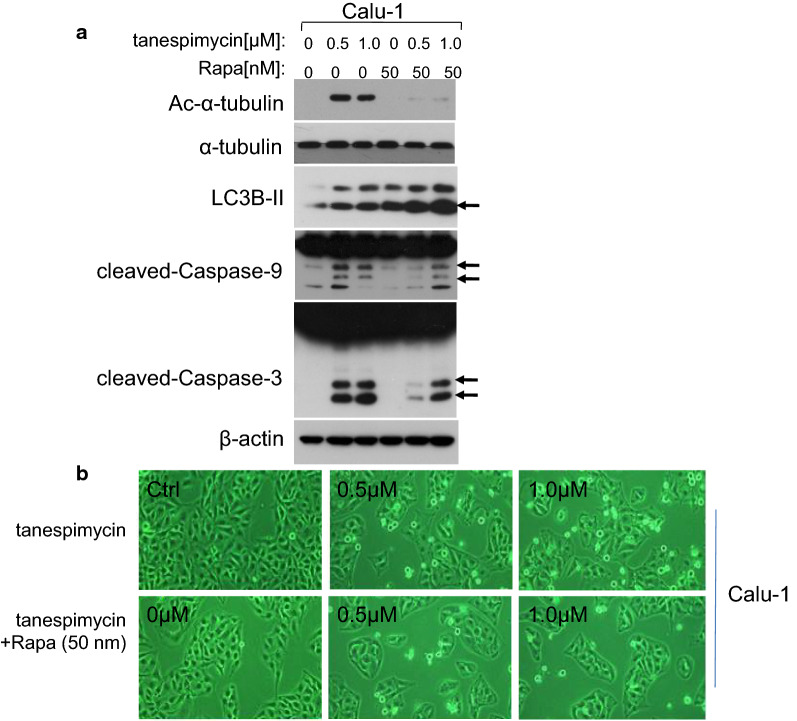
Rapamycin inhibits cell apoptosis induced by tanespimycin. Calu-1 cells were treated with tanespimycin (0, 0.5, 1.0 μM) or combination with Rapamycin (50 nM) for 48 h, and then (**a**), the whole-cell protein lysates were prepared and the given proteins (Ac-α-tubulin, α-tubulin, LC3B-II, cleaved-Caspase-9, cleaved-Caspase-3 and β-actin) were detected by Western blot analysis. **b** The cells were subjected to take photos by inverted phase contrast microscope to observe the survival state

Our previous experiments demonstrated that acetylation of α-tubulin was correlated with apoptosis and autophage. Which one of apoptosis and autophage is the predominant and whether the level of α-tubulin acetylation would be the determinant factor. So in order to testify the hypothesis, Calu-1 cells were treated by combination drugs of tanespimycin and Rapamycin, a well-known inhibitor of mTOR to induce autophage [30]. Experimental results showed that the expression of α-tubulin acetylation and cell apoptosis (evidenced by Caspase-3, Caspase-9 activation) were inhibited compared to tanespimycin treatment alone, but the level of LC3B-II was increased (Fig. 4a). In inverted phase contrast microscope, the amount of apoptotic cells of Calu-1 was reduced by combination of tanespimycin and Rapamycin. But in general, the number of living cells decreased, implying that cell division and apoptosis were inhibited at the same time (Fig. 4b). All these results showed that Rapamycin contributed to cell survival in the concentration of 50 nM in Calu-1 cells, and in this condition acetylation of α-tubulin was horizontal but LC3B-II expression progressively increased.

### 3-MA down-regulates LC3B-II and promotes cell apoptosis induced by tanespimycin

**Fig. 5:**
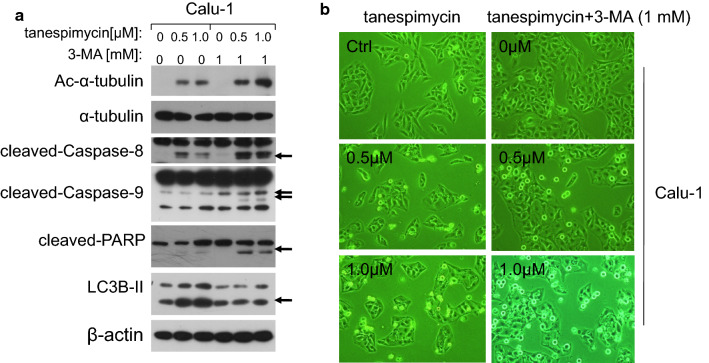
3-MA down-regulates LC3B-II and promotes cell apoptosis induced by tanespimycin. Calu-1 cells were treated with tanespimycin (0, 0.5, 1.0 μM) or combination with 3-MA (1 mM) for 48 h; **a** the cells were subjected to preparation of whole-cell protein lysates and the given proteins (Ac-α-tubulin, α-tubulin, cleaved-Caspase-8, cleaved-Caspase-9, cleaved-PARP, LC3B-II and β-actin) were detected by Western blot analysis. **b** the cells were subjected to take photos by inverted phase contrast microscope to observe the apoptotic state

Autophagy is a lysosomal pathway for degradation of organelles and proteins and it can provide metabolites and energy for cell survival. 3-MA is the inhibitor of autophage and promotes cell apoptosis [31]. In order to further detect the function of α-tubulin acetylation in autophage and apoptosis, tanespimycin was combined with 3-MA to investigate the expression of α-tubulin acetylation and LC3B-II in Calu-1 cells. At the concentration of 1 mM, 3-MA facilitated Ac-α-tubulin upregulation and apoptosis by combination with tanespimycin evidenced by activation of Caspase-8, Caspase-9 and PARP proteins which are hallmarks of apoptosis, but LC3B-II expression was reduced (Fig. 5a). In inverted phase contrast microscope, Calu-1 cells displayed moderate enhancement of apoptosis characteristics by combination of tanespimycin and 3-MA (Fig. 5b). From this experiment we further validated the correlation of α-tubulin acetylation between autophage and apoptosis.

### Z-vad inhibits apoptosis and up-regulates α-tubulin acetylation induced by tanespimycin

**Fig. 6:**
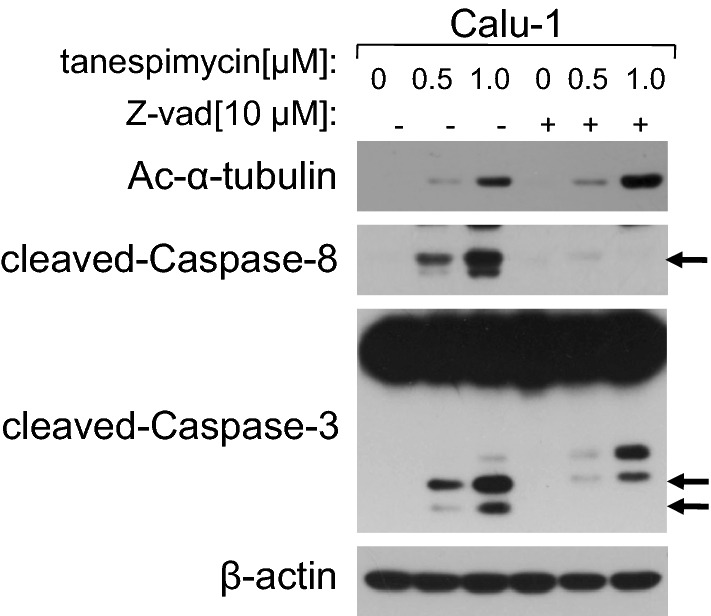
Z-vad inhibits apoptosis and up-regulates α-tubulin acetylation induced by tanespimycin. Calu-1 cells were treated with the given concentrations of tanespimycin (0, 0.5, 1.0 μM) or combination with Z-vad (10 μM) for 48 h, and then the cells were subjected to preparation of whole-cell protein lysates and the given proteins (Ac-α-tubulin, cleaved-Caspase-8, cleaved-Caspase-3 and β-actin) were detected by Western blot analysis

In the above experiments it has been investigated that acetylation of α-tubulin is implicated in apoptosis and autophage, and when the expression level of α-tubulin acetylation is increased substantially, the cell fate tends to apoptosis but not autophage. What determines cell fate for survival or apoptosis? In another words, the high expression level of α-tubulin acetylation is the cause or the subsequent consequence of apoptosis? In order to validate this hypothesis, Calu-1 cells were treated by combination of tanespimycin and Z-vad, an inhibitor of fan-Caspase proteins which inhibits apoptosis. At the concentration of 10 μM, Z-vad blocked cell apoptosis evidenced by partially inhibition of Caspase-3 activation, but the expression of α-tubulin acetylation was still increased moderately (Fig. 6), which implied that α-tubulin acetylation may the cause of apoptosis. It has also been found that the expression level of α-tubulin acetylation had a positive correlation with apoptosis, and when α-tubulin acetylation was highly induced, it would trigger apoptosis.

### Both HDAC6 and TAT1 modulate the expression levels of α-tubulin acetylation

**Fig. 7:**
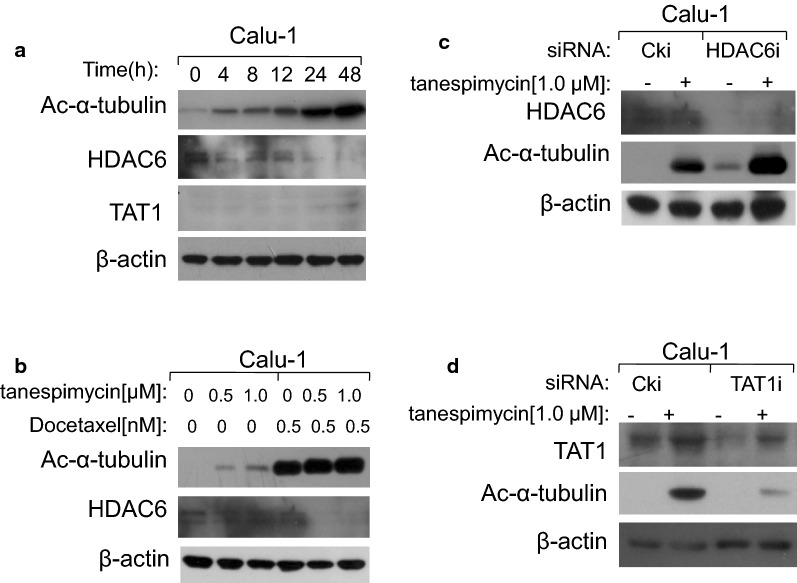
HDAC6 and TAT1 modulate the expression levels of α-tubulin acetylation in common. **a** Calu-1 cells were treated with 1.0 μM tanespimycin for the indicated time (0, 4, 8, 12, 24, 48 h). **b** Calu-1 cells were treated with the given concentrations of tanespimycin (0, 0.5, 1.0 μM) or combination with Docetaxel (0.5 nM) for 48 h. **c**, **d** Calu-1 cells were silenced by TAT1 siRNA and HDAC6 siRNA respectively, and then treated with 1.0 μM tanespimycin for 48 h. All the above cells were subjected to preparation of whole-cell protein lysates and the given proteins (**a** Ac-α-tubulin, HDAC6, TAT1 and β-actin; **b** Ac-α-tubulin, HDAC6 and β-actin; **c** HDAC6, Ac-α-tubulin and β-actin; **d** TAT1, Ac-α-tubulin and β-actin) were detected by Western blot analysis

**Fig. 8:**
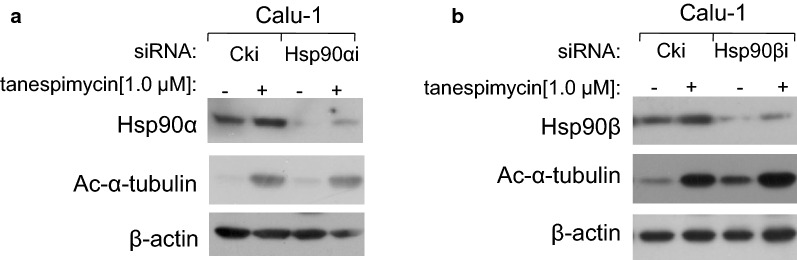
Hsp90 modulates Ac-α-tubulin expression level. Calu-1 cells were silenced by Hsp90 α/β siRNA respectively (**a**, **b**), and then treated with 1.0 μM tanespimycin for 48 h. All the above cells were subjected to preparation of whole-cell protein lysates and the given proteins (**a** Hsp90α, Ac-α-tubulin and β-actin; **b** Hsp90β, Ac-α-tubulin and β-actin) were detected by Western blot analysis

α-Tubulin and Hsp90 are two main non-histone substrates of HDAC6. When HDAC6 is inhibited, the acetylation levels of α-tubulin and Hsp90 are upregulated and their normal physiological functions may be changed. In our study it was demonstrated that when Hsp90 was disrupted by tanespimycin in time-course and dose-response experiments, HDAC6 expression levels were reduced and simultaneously α-tubulin acetylation was highly expressed (Fig. 7a, b), which indicated that HDAC6 was also the substrate of Hsp90, and this result was consistent with what has been reported in a recent literature [16]. Perhaps there may be a positive modulatory loop between Hsp90 and HDAC6. Besides the deacetylase HDAC6, acetyltransferase TAT1 is the reported α-tubulin acetyltransferase in mammalian [19]. In order to testify whether TAT1 and HDAC6 are the two enzymes that modulate the expression of α-tubulin acetylation in Calu-1 cells induced by tanespimycin, TAT1 and HDAC6 were silenced by RNA interference technique respectively to detect the expression of α-tubulin acetylation. Apparently, when TAT1 or HDAC6 was silenced, the expression level of α-tubulin acetylation was reduced or increased, suggesting that TAT1 was the acetyltransferase and HDAC6 was the deacetylase of α-tubulin in Calu-1 cells, and they regulated Ac-α-tubulin expression in common (Fig. 7c, d). In Hsp90α/β knockdown experiments, it was found that when Hsp90α/β was silenced by siRNA, the expression of Ac-α-tubulin was also increased, which indicates that Hsp90 modulates the expression of α-tubulin acetylation (Fig. 8a, b).

### Tanespimycin induces Hsp90α/β and Hsp27 upregulation

**Fig. 9:**
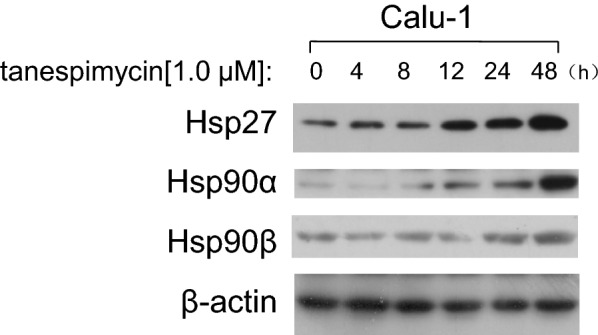
Tanespimycin induces Hsp90α/β and Hsp27 up-regulation. Calu-1 cells were treated with 1.0 μM tanespimycin for the indicated time (0, 4, 8, 12, 24, 48 h), and then the cells were harvested for preparation of whole-cell protein lysates for following Western blot analysis to detect Hsp90α, Hsp90β and Hsp27 levels

As the molecular chaperone, Hsp90 is predominantly associated with misfolded proteins ubiquitination and degradation. And other Heat shock proteins, such as Hsp70, Hsp27 and so on, participate in protein folding, protein translocation and transportation, and they also function as molecular chaperones to help and guide misfolded proteins refolding or by assisting their delivery to the ubiquitin/proteasome degradation system, and thus have an important role in safeguarding stressed cells [32, 33]. In our study it was investigated that in Calu-1 cells, the expression levels of Hsp90α or Hsp90β were increased in time-dependent manner (Fig. 9) and consistent with the expression of α-tubulin acetylation. Hsp27 protein was increased more apparent (Fig. 9). Collectively, it was concluded that the dual functions of acetylation of α-tubulin in Calu-1 cells are dependent on Hsps expression.

## Discussion

Cell survival and apoptosis are modulated by variety of factors including cell intrinsic balance and extrinsic inducing factors. Programmed cell death and autophage are the two main pathways for degrading misfolded or unnecessary proteins to maintain whole organism healthy growth and survival. At the onset of outer stimuli, cells would trigger autophage or apoptosis. Most of the anti-tumor drugs induce cancer cell apoptosis via different apoptosis pathway or activate one/some key proteins to display anti-tumor efficacy. Tanespimycin is a well known anti-tumor drug which has been testified in II or III clinical trials. Studies have elucidated that tanespimycin leads to protein degradation by autophage or apoptotic induction [4, 5]. In our study tanespimycin induces cell apoptosis and autophage simultaneously evidenced by activation of Caspase proteins and LC3B-II, which are the markers of apoptosis and autophage respectively. Furthermore, the degree of cell apoptosis is correlated with levels of α-tubulin acetylation. In our experiments, autophage is enhanced along with the increasing of α-tubulin acetylation, but when the expression of α-tubulin acetylation reaches a certain level, autophage is inhibited and apoptosis is facilitated. All these results indicated that acetylation level of α-tubulin may be a determinant factor for cell death and survival and may be in the crossroad involving cell apoptosis and autophage.

It has been well known that microtubules play key roles in many cell physiological functions such as cell division, cell motility, intracellular trafficking, cell signaling transduction and so on. As one of the two main components of microtubules, α-tubulin and its acetylation affect microtubule properties and functions and consequently affect cell fate [34]. Our study results show that Hsp90 inhibition evokes acetylation of α-tubulin further. In this respect acetylation of α-tubulin would promote autophage clearance evidenced by activation of LC3B-II. It has been reported that Palitaxel induces cell apoptosis and autophage in cancer cells [35]. In our combination adminstration study, Docetaxel also simultaneously evokes autophage by activation LC3B-II and α-tubulin acetylation. But cells undergo to apoptosis not autophage following α-tubulin acetylation in quantity by combination administration of Docetaxel and tanespimycin. In order to maintain normal microtubule functions, we speculate the total acetylation level of microtubule proteins must be at the “threshold value”. If the acetylation level of α-tubulin exceeds the “threshold value”, microtubule properties and functions would be changed and they convert to induce cell apoptosis.

Regard to the roles of autophage in cancer therapy, there has been conflicting views in previous studies. Autophagy is considered as a protective mechanism against cell apoptosis under starvation condition and is also contributed to resist against chemotherapeutic-induced cell death in cancer cells [36]. But on the other hand, there is emerging reports that autophagy plays important roles in the generation of anticancer responses and mediates malignant cell death [37–39]. In previous studies, many frontline anticancer agents induce cell apoptosis via diverse molecular mechanism of action and they would be predicted to stimulate autophagy or apoptosis [40–42]. Our studies have also found that by combination of TSA, tanespimycin induces cell apoptosis and autophage following acetylation of α-tubulin in quantity and activation of LC3B-II. In these results autophage induced by tanespimycin and TSA in Calu-1 cells is Caspase-dependent.

In order to validate the function of α-tubulin acetylation in cell apoptosis and autophage, Rapamycin or 3-MA was combined with tanespimycin respectively in Calu-1 cells. Rapamycin is the inhibitor of m-TOR and promotes autophage, while 3-MA is the inhibitor of autophage. In above two experiments, we found that the level of α-tubulin acetylation was consistent with the degree of cell apoptosis. It is worthy to note that in this condition cell apoptosis was apparent and Caspases activation and PARP cleavage were strong. Our experiments have also investigated moderate acetylation of α-tubulin was contributed to autophage. In this case the level of α-tubulin acetylation was consistent with activation of LC3B-II. But when the expression level of α-tubulin acetylation exceeded the “threshold value”, the fate of cell was only to apoptosis (LC3B-II was inhibited). This conclusion has been testified when Caspases activation was inhibited by Z-vad, the level of α-tubulin acetylation was still increased at the presence of tanespimycin, which indicated that acetylation of α-tubulin emerged previously and apoptosis subsequently.

Deacetylase HDAC6 and Histone acetyltransferase (HAT) modulate the acetylation level of α-tubulin reported in literatures. Indeed, our studies have also discovered when Hsp90 was inhibited by agents or silenced by siRNA, the expression of HDAC6 was reduced and the level of α-tubulin acetylation was increased. Hsp90 inhibition evoked HDAC6 decrease and Ac-α-tubulin increase, implying that HDAC6 is also the substrate of Hsp90, and there exists a regulatory loop between Hsp90 and HDAC6. This was also testyfied by Hsp90α/β knockdown experiments, in which the expression of Ac-α-tubulin was increased. Our study results also confirmed that HDAC6 and TAT1 modulated the acetylation of α-tubulin by siRNA technique in calu-1 cells.

## Conclusions

According to our previous experiments and results, it is concluded that the level of α-tubulin acetylation has a dual functions in apoptosis and autophage induced by Hsp90 inhibitor tanespimycin in Calu-1 cells. At a lower level of α-tubulin acetylation, cells are inclined to autophage in a Caspase-dependent manner. But with the increase of α-tubulin acetylation, cells convert to apoptosis and cellular autophage is attenuated. How to determine the “threshold value” of α-tubulin acetylation and the agent concentration to induce cell apoptosis effectively in cancer therapy is to further study.

## Data Availability

All data in this study are present in the published article. The datasets analyzed during this research are available for scientific study from the corresponding author on reasonable request.

## Abbreviations

Ac-α-tubulin: Acetylated α-tubulin
HDAC6: Histone deacetylase 6
Hsp90: Heat shock protein 90
Hsp27: Heat shock protein 27
LC3B-II: Microtubule-associated protein 1 light chain 3B-II,
PARP: Poly ADP-ribose polymerase
SRB: Sulforhodamine B
TAT1: α-Tubulin acetyltransferase 1
TSA: Trichostatin A
Z-vad: Caspase inhibitor
3-MA: 3-Methyladenine
